# Craniofacial Analysis May Indicate Co-Occurrence of Skeletal Malocclusions and Associated Risks in Development of Cleft Lip and Palate

**DOI:** 10.3390/jdb8010002

**Published:** 2020-01-28

**Authors:** Denise K. Liberton, Payal Verma, Konstantinia Almpani, Peter W. Fung, Rashmi Mishra, Snehlata Oberoi, Figen Ç. Şenel, James K. Mah, John Huang, Bonnie L. Padwa, Janice S. Lee

**Affiliations:** 1 Craniofacial Anomalies and Regeneration Section, NIDCR, NIH, Bethesda, MD 20892-1470, USA; denise.liberton@gmail.com (D.K.L.); nadine.almpani@nih.gov (K.A.); 2 Division of Oral & Maxillofacial Surgery, University of Cincinnati, Cincinnati, OH 45267-0558, USA; payal.verma@me.com; 3 Department of Oral & Maxillofacial Surgery, University of North Carolina, Chapel Hill, NC 27599-7450, USA; pfung87@gmail.com; 4 Division of Oral Medicine, University of Washington, Seattle, WA 98195-6370, USA; rashmi1402@gmail.com; 5 Orofacial Sciences, University of California, San Francisco, CA 94143, USA; Sneha.Oberoi@ucsf.edu; 6 Health Institutes of Turkey, Turkish Healthcare Quality and Accreditation Institute, Istanbul 34718, Turkey; fcsenel@hotmail.com; 7 Advanced Education Program in Orthodontics and Dentofacial Orthopedics, School of Dental Medicine, University of Nevada, Las Vegas, NV 89154, USA; james.mah@unlv.edu; 8 Private Practice Orthodontist, San Leandro, CA 94577, USA; drjohnhuang@yahoo.com; 9 Harvard School of Dental Medicine, Oral Surgeon in Chief, Department of Plastic and Oral Surgery, Boston Children’s Hospital, Boston, MA 02115, USA; bonnie.padwa@childrens.harvard.edu

**Keywords:** non-syndromic cleft, craniofacial morphology, midfacial growth, maxilla, mandible, malocclusion, cephalometry, computerized tomography

## Abstract

Non-syndromic orofacial clefts encompass a range of morphological changes affecting the oral cavity and the craniofacial skeleton, of which the genetic and epigenetic etiologic factors remain largely unknown. The objective of this study is to explore the contribution of underlying dentofacial deformities (also known as skeletal malocclusions) in the craniofacial morphology of non-syndromic cleft lip and palate patients (nsCLP). For that purpose, geometric morphometric analysis was performed using full skull cone beam computed tomography (CBCT) images of patients with nsCLP (*n* = 30), normocephalic controls (*n* = 60), as well as to sex- and ethnicity- matched patients with an equivalent dentofacial deformity (*n* = 30). Our outcome measures were shape differences among the groups quantified via principal component analysis and associated principal component loadings, as well as mean shape differences quantified via a Procrustes distance among groups. According to our results, despite the shape differences among all three groups, the nsCLP group shares many morphological similarities in the maxilla and mandible with the dentofacial deformity group. Therefore, the dentoskeletal phenotype in nsCLP could be the result of the cleft and the coexisting dentofacial deformity and not simply the impact of the cleft.

## 1. Introduction

Non-syndromic cleft lip and cleft palate (nsCLP) is one of the most common congenital craniofacial abnormalities [1,2]. Etiologically, multiple genes appear to influence the risk for nsCLP but differ across ethnic populations and families. Exome and genome-wide association studies, as well as candidate gene sequencing studies, have investigated the effects of various genes, rare gene variants, and gene modifiers in the occurrence of nsCLP [3,4,5,6,7,8,9,10,11,12,13,14,15,16,17,18,19,20]. Additionally, there is evidence that environmental factors have a role in the etiology of nsCLP and specific gene-by-environment interactions have been identified [20,21]. Dentofacial deformities, also known as skeletal malocclusions, and defined as malocclusions associated with abnormal skeletal growth patterns that are not syndromic, have also been found to be under strong genetic control with an additional environmental influence [22,23,24,25,26,27,28] and often co-exist with nsCLP [29,30,31,32].

Some studies suggest that there is an intrinsic maxillary growth deficiency in patients with nsCLP [33,34], while other studies suggest facial growth may be affected by early surgical interventions for cleft lip and/or palate repair, creating lip tension and scar tissue surrounding the palate and maxilla. To date, the debate still persists. These surgical procedures have frequently been reported to induce posterior displacement of the maxilla in patients with nsCLP [35,36,37,38,39], with variable magnitude that depends on the amount of tissue deficiency, the surgical protocol, and the skill of the surgeon [40,41,42,43]. Procedures such as gingivoperiosteoplasty, a surgical method that tunnels and connects the gingiva around the anterior maxilla by the alveolar cleft, performed around 3 months of age to avoid secondary bone grafting, has been shown to restrict maxillary anterior growth and has fallen out of favor [44]. In general, extensive surgical procedures with more significant scarring [45] and palatal surgery seem to have more significant influence on the growth of the midface than lip surgery. 

The craniofacial morphological changes in the presence of a cleft are obvious and are the reason that many patients with nsCLP require secondary facial reconstructive surgery once growth is complete. The focus has been primarily on the development of the maxilla with little evaluation of the overall craniofacial development (mandible and cranial base), despite the fact that many cephalometric analyses studies demonstrate that both the maxilla and mandible are abnormal in growth [46]. These observations begin to suggest a more global relationship between craniofacial development and clefts. However, it is unclear how the underlying inherent dentofacial deformity may actually impact craniofacial development in the setting of a cleft and whether the dentofacial deformity or the presence of a cleft increases the propensity for the other. 

Non-syndromic dentofacial deformities are common developmental growth anomalies that manifest during puberty, which affect approximately 13% of the US population [47]. The classification of dentofacial deformities is based on the relationship of the maxilla and mandible to each other and to the cranial base. Individuals with a normal occlusion and normal relationship of the maxilla and mandible to each other have a straight facial profile and are considered Class I. Class II skeletal malocclusion often includes mandibular teeth that are positioned too posterior to the maxillary teeth (deep overbite) and associated with a retrognathic (“weak”) mandible; this occurs in approximately 10% of the population and 1% will require surgical correction. Class III skeletal malocclusion manifests with mandibular teeth that are positioned in front of the maxillary teeth (underbite) and associated with a prognathic mandible or a hypoplastic maxilla or both. This is not as common and is seen in nearly 3% of the population; however, surgical correction is necessary in 40% of this group. These deformities impact function and the more severe manifestations of these dentofacial deformities require skeletal correction with orthognathic surgery to re-establish the balance of the craniofacial structures while the milder manifestations that are localized to the teeth may be treated with orthodontic therapy only. The Class III dentofacial deformity and underbite are seen more often in patients with nsCLP. This requires additional surgery for patients with clefts once skeletal maturity has been achieved.

In previous studies, two-dimensional cephalometric radiographs were mainly used for the analysis of sagittal growth and transverse morphology in patients with nsCLP. More recent studies have used three-dimensional photos [48] or cone-beam computed tomography (CBCT) [49,50,51]. However, regardless of the type of cephalometric data, previous studies have evaluated nsCLP patients using either existing two-dimensional normative cephalometric data for the general population or matched healthy control groups [49,50,51,52,53]. These previous studies have included unaffected individuals without clefts as controls, with very little skeletal information. However, it should be clear that the absence of a cleft does not mean that craniofacial skeletal development is “normal.” The contribution of any co-existing dentofacial deformity to the general craniofacial phenotype in patients with nsCLP is dismissed when controls or normal individuals are defined as simply those without a cleft, and the deviation of these patients’ morphology from the norm is attributed entirely to the existence of the cleft defect and/or the impact of surgical intervention.

The purpose of this study is to determine whether and in what way dentofacial deformities (skeletal malocclusions) affect the craniofacial shape and growth in patients with nsCLP. To achieve this, the craniofacial morphology of a cohort of patients with nsCLP who underwent cleft repair was compared to two different control groups consisting of unaffected Class I as well as matched Class II or Class III skeletal malocclusion subjects, depending on their co-existing dentofacial deformity. The craniofacial analysis was based on anatomical landmarks annotated on three-dimensional CBCT images. A multivariate geometric morphometric analysis of the landmark coordinates was conducted and several observations regarding the craniofacial morphology of patients with nsCLP were made based on these results.

## 2. Methods

### 2.1. Subjects

This is a retrospective, cross-sectional study that included a total of 120 subjects. Thirty subjects were patients with non-syndromic cleft lip and palate (nsCLP) who had undergone cleft repair but had not yet undergone additional orthognathic surgery. Each subject with nsCLP was compared to three control subjects: two with normal occlusion (unaffected controls) as well as one with the comparable skeletal malocclusion. The first control group included 60 skeletal Class I patients, with normal bite and without any craniofacial deformities, known henceforth as the “unaffected” control group. The second control group included 30 Class II and III skeletal malocclusion patients, without any other craniofacial deformities, known henceforth as the “dentofacial deformity” (DFD) group. Because malocclusions may occur for isolated dental anomalies, we excluded those who had malocclusion without the corresponding skeletal cephalometric changes (see below). All CBCT images were initially acquired for clinical care purposes and were included in this study for secondary data analysis. The control groups’ database includes images taken on individuals that were either candidates for or undergoing orthodontic treatment (unaffected control group) or candidates for orthognathic surgery to address a skeletal malocclusion (DFD group).

The dentofacial deformities were classified based on the dental malocclusion classification (Angle’s class) in combination with sagittal skeletal cephalometric measurements: the Sella point to Nasion to A point (SNA), the Sella point to Nasion to B point (SNB) and the A point to Nasion to B point (ANB) angles (Supplementary Table S1). The ANB angle expresses the sagittal intermaxillary relationship and was also used to match each control subject as closely as possible to the specific ANB measure of a cleft patient of the same sex after they were initially matched based on their malocclusion category. For the general population, the mean value for ANB is 2° with a standard deviation of ±2° [54]. In our cleft cohort, the mean ANB for Class II subjects was 4.54° (Range: 1.40°–10.82°, SD ±2.77°) and the mean ANB for Class III subjects was −5.64° (Range: −13.40°–2.15°, SD ±2.77°). For some patients with nsCLP, the ANB was significantly more extreme than any comparative sample in the DFD cohort. For our Class I control subjects, the mean ANB was 3.37° (Range: 0.82°–5.05°, SD ±4.24°), for Class II DFD controls the mean ANB was 5.09° (Range: 1.46°–7.89°, SD ± 2.10°), and the mean ANB for Class III patients was −4.19° (Range: −7.42°–0.57°, SD ±2.25°). Despite our best effort to identify and compare patients of similar age, specific treatment requirements necessarily led to a few age differences among the cohorts. In particular, the mean age of the cleft group was 17.6 years, with a range from 9 to 28 years, whereas the mean age of the unaffected control group was 16.0 years, with a range of 13 to 24 years. The younger ages in the cleft group are justified by the fact that the starting age for orthodontic treatment is normally earlier than the average, due to the need for palatal expansion in combination with maxillary advancement, alleviation of maxillary crowding or preparation for alveolar bone grafting. The mean age of the DFD group was 22 years, with a range from 13 to 38 years. The DFD patients were significantly (*p* < 0.05) older than the other cohorts, as most of these individuals were orthognathic surgery candidates, which is not performed until skeletal maturity. In addition, the best effort was made to match the groups with regard to ethnicity. Information on the structure of the sample including diagnosis, age, sex, skeletal class, cephalometric measures, cleft type, and laterality is shown in Table 1.

Their de-identified records and CBCT scans were obtained from the University of California San Francisco (UCSF), Boston Children’s Hospital, and the University of Nevada Las Vegas. The study was conducted in accordance with the Declaration of Helsinki through IRB-approved protocols and data-sharing agreements between the respective institutions and the NIDCR investigators (NIDCR IRB #16-D-0040, UCSF MTA #T-2014-2541; BCH IRB #X05-08-058, DSA #T-2016-3598; UNLV IRB #1002690-1, DSA #T-2016-3596). All records were evaluated by an orthodontist (KA) and three oral and maxillofacial surgeons (PV, JSL, FS) and verified for skeletal classification and diagnosis.

### 2.2. CBCT

**Figure 1:**
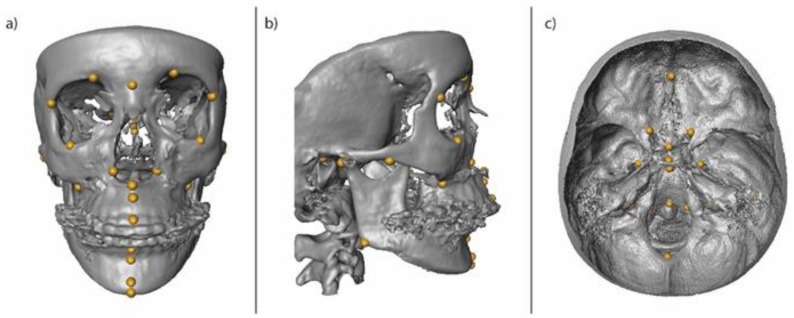
Volume rendering of a 3D skull in a frontal (**a**), lateral (**b**), and axial section (**c**) view of the cranial base, including the anatomical landmarks that were annotated on the CBCT images and used for the morphometric analysis. Each landmark is indicated by a yellow dot [55]. The list of landmarks can be found in Supplementary Table S2.

Full skull CBCT scans were obtained via a CB MercuRay system (Hitachi Medical Corporation, Tokyo, Japan) at UCSF and UNLV at 0.377 mm voxel size, and a Planmeca Promax 3D Max system (Planmeca, Helsinki, Finland) at Boston Children’s Hospital at 0.300 mm voxel size. 3D reconstructions were performed in Invivo 5.4 (Anatomage, San Jose, CA) and 37 craniofacial landmarks (Supplementary Table S2) were annotated on each CBCT scan by a single researcher with expertise in 3D cephalometric analysis (PV) (Figure 1). The 3D landmarks were selected from a larger set of 61 standardized landmarks with low intra- and inter-rate errors to provide coverage across the craniofacial complex and provide thorough information regarding the vertical, transverse, and sagittal relationships of the craniofacial structures as well as the degree of asymmetry for each subject [55]. Landmark XYZ coordinates were exported from Invivo as .csv files and merged using a custom Perl script for subsequent shape analysis. Prior to the conduction of the shape analysis, individuals with unilateral clefts on the left side were mirrored by inverting the sign of all X coordinates and landmark labels were exchanged so that all unilateral clefts could be visualized on the right side of the skull. This step was required for the reduction of noise from the random distribution of cleft laterality.

Landmark coordinates were imported into R software [56] for statistical analysis and analyzed via geometric morphometrics using the geomorph package [57,58]. A total of 22 landmarks across all individuals (0.495% of all landmarks) were unable to be localized due to limitations in the fields of view in these particular scans. These landmarks included the cribriform plate, opisthion, frontozygomatic suture, and anterior cranial fossa. None of the missing landmarks were near the midface and thus were less likely to vary in position due to the presence of a cleft. Missing landmarks were imputed via TPS using the estimate.missing function. Procrustes superimposition was performed without object symmetry to preserve the asymmetric effects of unilateral clefts on craniofacial shape. Procrustes superimposition involves rotation, translation, and scaling to standard centroid size and produces a set of Procrustes coordinates. As our sample included adolescents and adults, we adjusted for the effects of ontogeny by performing a multivariate linear regression of the Procrustes coordinates against subject age to remove the age-associated variation in facial shape. We then performed a second multivariate regression on the age residuals against centroid size to control for the effects of allometry or overall craniofacial size on craniofacial shape. Significance values were obtained via permutation testing (1000 permutations). The residuals of these regressions, called Procrustes residuals, were used for all subsequent analyses.

First, we explored the facial shape variation in the complete dataset of unaffected controls, DFD, and nsCLP subjects via principal component analysis (PCA), which is a multidimensional data reduction technique that uses eigenvalue decomposition of the landmark covariance matrix to determine the axes in multidimensional space that maximize the within-sample variation. The results of the PCA are presented in principal component (PC) scores, values representing individuals in the transformed space, which were plotted to show the extent of morphological variation and clustering across the sample. It also provides loadings or eigenvectors associated with each PC that indicate the weighted contribution of individual landmarks to a given axis. Next, a Procrustes ANOVA was performed on the Procrustes residuals to test for significant differences in craniofacial shape among the sexes and diagnoses (cleft, unaffected control, and dentofacial deformity). Significance for Procrustes ANOVA was assessed via permutation testing (1000 permutations). Further permutation testing (10,000 permutations) was performed to test for significant differences in the consensus or mean craniofacial shapes of each group by Procrustes distance and was used to perform pairwise comparisons and localize shape differences between the groups that were found to be significant in the Procrustes ANOVA. We also performed bootstrapping analysis (1000 replicates) on the trace of the covariance matrix within each group to obtain an overall measure of shape variance for each group. ANOVA was used to assess significance difference in variance among the groups.

A second analysis was performed only on the nsCLP group to investigate the effects of the type of cleft (unilateral or bilateral) on craniofacial shape variation among individuals with clefts. In this analysis, because of the small sample size in the nsCLP group, we did not have sufficient power to look at the interaction between cleft type (unilateral and bilateral) and skeletal malocclusion (Class II or Class III). Therefore, we statistically removed the effects of skeletal malocclusion from shape data. First, we calculated the mean difference for all landmark coordinates between the cleft Class II and Class III groups. We manually added this difference to the coordinates of all individuals of the cleft Class II group, adjusting their mean shape to that of the cleft Class III group. Post-adjustment, no significant effects of skeletal malocclusion were found within the nsCLP group (*p* = 1.000) and all further analysis reflects the effects of cleft type on craniofacial morphology rather than skeletal class. A second PCA was performed on the adjusted coordinates to examine shape variation and permutation testing was used to test for significant effects of cleft type on craniofacial shape by calculation of the Procrustes distance between the unilateral and bilateral cleft groups.

## 3. Results

### 3.1. Ontogeny, Allometry and Sexual Dimorphism

The results of the multivariate regression analysis revealed a significant effect of age on craniofacial shape (*p* = 0.002) and that age explained 2.11% of the variation in shape in the combined sample of nsCLP, DFD, and unaffected controls. A second multivariate regression analysis on the age-residuals showed that centroid size was also significant (*p* = 0.002) and explained an additional 3.91% of the residual shape variation. Combined, age and size can, therefore, explain 6.02% of craniofacial shape, indicating that craniofacial shape does change with age and craniofacial size. This was an expected result, given the fact that many of our subjects had not yet reached adulthood and reinforces the importance of controlling for the effects of age and size on craniofacial shape. Using Procrustes ANOVA on the age and size regression residuals, we also tested for the existence of sex differences, but no significant effects of sex on craniofacial shape (*p* = 0.732) were detected in this sample. While there are known differences in the shape of the skull due to sex, many features can be attributed to differences in growth and body size and therefore we did not consider any additional effects of sex in this cohort.

### 3.2. Shape Variation in the Combined Sample (CLP, DFD and Unaffected)

**Figure 2:**
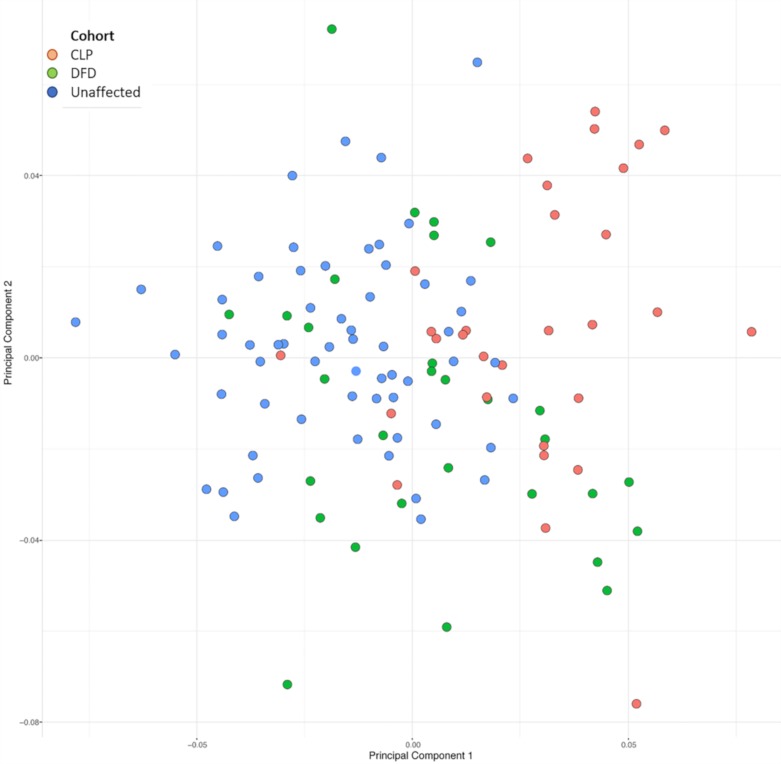
Plot of the first two PC axes from the principal component analysis, which explains 29.6% of the shape variance in the combined sample of patients including the unaffected controls (Class I), DFD, and nsCLP. Using PCA, we are able to demonstrate the overall craniofacial shape differences between the three groups, with the DFD group having overlap with the unaffected controls and the nsCLP group. The fact that there is little overlap between the unaffected controls and the nsCLP indicates that, while the groups were age- and gender-matched, the overall clusters are distinct in craniofacial shape and skeletal variances. The cohorts are indicated by color of the dot.

According to the results of the principal components analysis, the first 30 principal components (PCs) out of 104 total explain 90% of the overall craniofacial shape variation in the combined sample, with the first three PCs explaining over 41%. A plot of the first two PCs can be found in Figure 2. There is an overlap between the DFD group and both the unaffected controls and cleft groups on PC1, indicating that individuals with isolated DFD have a variable and intermediate craniofacial morphology compared to the other groups. However, the cleft and unaffected control groups cluster separately from each other, with little overlap, indicating greater differences in craniofacial shape. PC2 did not show any clustering according to cohort.

PC1 explains 16.38% of the overall craniofacial variance and is associated with variation in the overall width and height of the skull, with the positive axis indicating shorter, narrower features than the negative axis. Additionally, PC1 is associated with severe maxillary hypoplasia and greater flexion in the cranial base along the positive axis and normal maxillary growth and decreased flexion of the cranial base along the negative axis. The most significant variation for this axis occurs in the maxilla and palatal regions, with the nasal cavity, A point, ANS and PNS landmarks showing the greatest displacements along this axis with shorter maxillary and palatal lengths on the positive axis. PC2 explains 13.22% of the craniofacial variation and the facial shape changes are associated with jaw projection. Additionally, it affects the upper facial and cranial base width, as well as the bicondylar jaw width, but not the width of the lower part of the jaw. PC3 explains an additional 11.39% of the variance and is associated with rotational changes in the gonial angle of the mandible. Along the positive axis of PC3, the mandible rotates down and backwards along the positive axis and forward and upwards along the negative axis.

Procrustes ANOVA, a multivariate ANOVA using the shape coordinates, supported the PCA results and revealed significant differences in shape among the three groups (nsCLP, DFD, and unaffected control), with diagnosis explaining 10.70% of the variation in the combined sample (*p* = 0.001). When we used a more complex linear model that included diagnosis, skeletal class, and the interaction term, we found that skeletal class explains an additional 4.61% of craniofacial shape variation (*p* = 0.001) and the interaction term explains an additional 1.53% of the variation (*p* = 0.028). In total, this model explained 16.84% of craniofacial shape variation among the nsCLP, DFD, and unaffected controls cohorts. Pairwise Procrustes distance (PD) comparisons, which are a measure of overall shape difference between groups based on the difference between mean landmark configurations, indicated that all three diagnoses differ significantly in terms of overall craniofacial shape (*p* < 0.0001 for all comparisons), with the nsCLP group differing the most from the unaffected controls (PD = 0.054) and slightly less so from the DFD cohort (PD = 0.044). The DFD cohort and unaffected controls were the most similar to each other (PD = 0.039). Overall, the DFD group had the highest shape variance (trace = 0.00514), followed by the nsCLP group (0.00513), while the unaffected controls had the lowest shape variance (0.00508).

**Figure 3:**
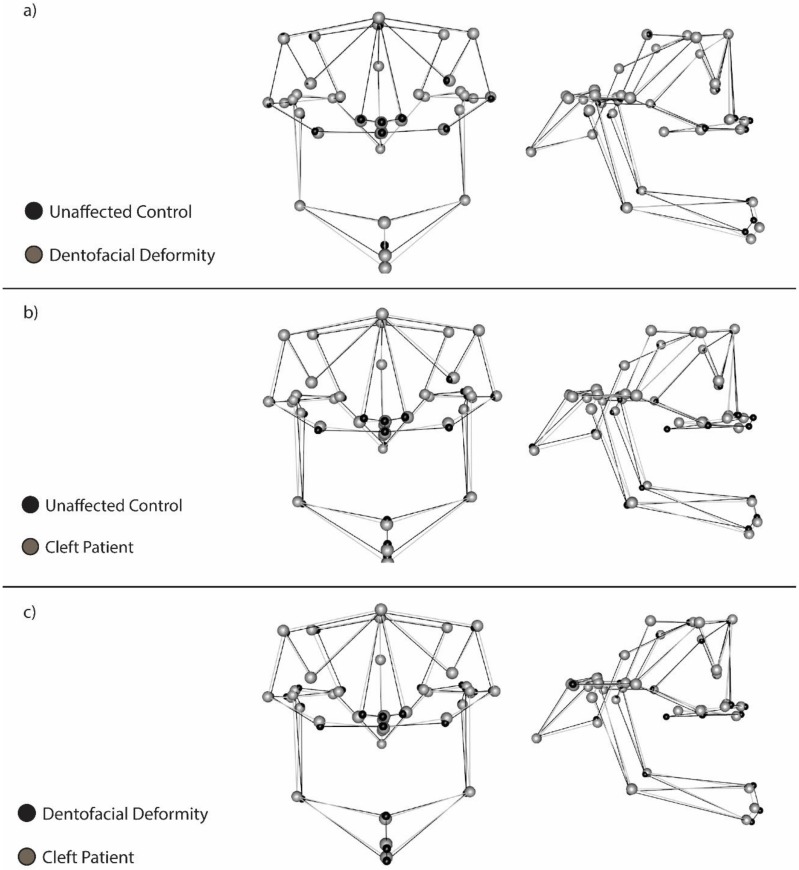
Pairwise wireframe overlays comparing mean craniofacial shape of each cohort. Frontal and sagittal views are presented. All pairwise comparisons are shown: (**a**) unaffected controls compared to patients with dentofacial deformities; (**b**) unaffected controls compared to patients with clefts; (**c**) patients with dentofacial deformity compared to patients with clefts. Both dentofacial deformity and nsCLP cohorts vary from the unaffected control group in a similar fashion (**a**,**b**) with maxillary hypoplasia/retrognathia and mandibular hyperplasia/prognathia. The third wireframe, (**c**), shows that the dentofacial deformity and nsCLP group are highly comparable in shape despite the presence of a cleft in one group. The mandible shape is also affected in the nsCLP. It is important to note that the maxilla in the nsCLP is deficient even in areas distant from the cleft site, i.e., right and left jugal points and is generally more hypoplastic but wider than the dentofacial deformity group. Landmarks are indicated by dots and a greater distance between dots in the two cohorts indicates differences in shape at that location between the two cohorts. The lines connecting dots are provided to better indicate overall skull shape and three-dimensionality.

Pairwise comparisons of the mean craniofacial shape among cohorts are shown in Figure 3. Compared to the unaffected controls, the cleft group presented with a degree of maxillary hypoplasia, as indicated by the more posterior position of landmarks such as the A point, the anterior nasal spine, the right and left corners of the nasal cavity, and the left and right jugal points. The posterior nasal spine is positioned more anteriorly, indicating an overall shortening of the palate in the cleft group. The gonial angle was larger in the cleft group and the landmarks on the medial plane of the mandible (B point, pogonion, and menton) were located more anteriorly and inferiorly than in the unaffected controls group. In the transverse plane, the cleft group presented slightly wider distances between all bilaterally located landmarks.

Compared to unaffected controls, in the DFD group, the A point, the anterior nasal spine, the left and right nasal cavity and the jugal points are more posteriorly located. However, the differences in the position of these landmarks are smaller in magnitude than those seen between the cleft and unaffected control groups. There is no difference in the location of the posterior nasal spine between these groups, indicating that the shorter palate is unique to the cleft group and is not corrected by cleft palate repair. The B point, pogonion, and menton landmarks were located more anteriorly and inferiorly in the DFD group than in the unaffected control group, likely because of the prognathic patients in the DFD group. For these landmarks, there was a greater difference between the DFD and unaffected control group than between the cleft and control group, indicating a greater skeletal discrepancy associated with a prognathic profile in the mandible of DFD patients than in the patients with clefts. There was also no significant difference in the position of landmarks from a transverse perspective, indicating similar skeletal width measures in both DFD and unaffected control groups. Finally, compared to the cleft group, the DFD group presented with a smaller magnitude for all transverse features. In the midface, while the same landmarks altered in both groups compared to the unaffected controls, there is even greater midface hypoplasia in the cleft group. The A point, anterior nasal spine, the nasal cavity width landmarks, and the jugal points were more posteriorly located while the posterior nasal spine was more anteriorly located, indicating a shortening of the midface from both the front and back. In terms of the mandible, the differences in mandibular prognathism in the DFD group indicate a greater degree of Class III skeletal malocclusion in comparison to the cleft group, which is consistent with the findings from comparisons of either to the unaffected control group.

A second pairwise Procrustes distance analysis similarly revealed significant pairwise shape differences between the main three skeletal malocclusion types: Class I, Class II, and Class III (*p* < 0.0001 for all comparisons). Class I and Class II were the least different in terms of overall shape (PD = 0.038), while Class III showed differences compared to both Class I (PD = 0.050) and Class II (PD = 0.050). 

The trace of the covariance matrix was calculated as a measure of variance within each diagnostic group. The variance was highest in the CLP group (mean trace = 0.00588), followed by the DFD group (mean trace = 0.00536), and the variance was lowest in the unaffected controls (mean trace = 0.00401). ANOVA found significant differences among the groups (*p* < 0.0001) and pairwise comparisons were all significant (*p* < 0.0001) (Supplementary Figure S1).

### 3.3. Shape Variation within the CLP Sample (Unilateral and Bilateral)

**Figure 4:**
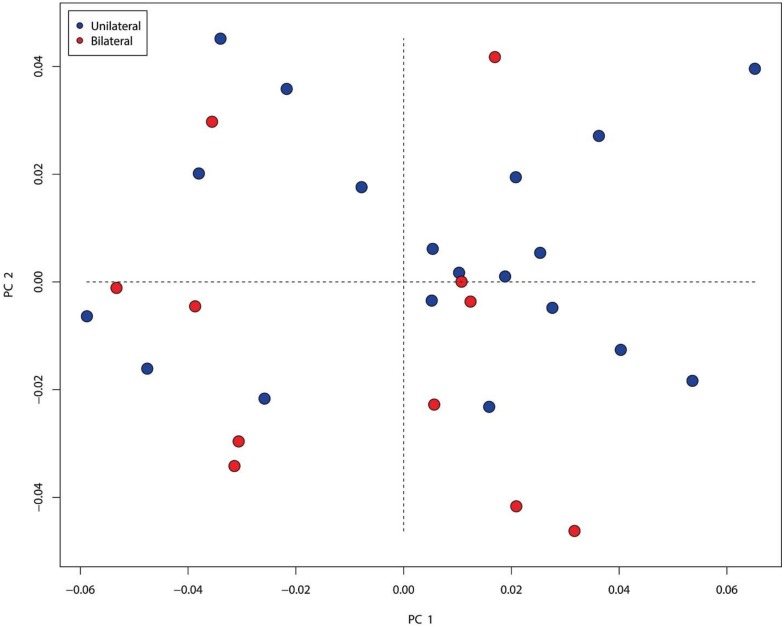
Plot of the first two PC axes from principal component analysis, which explains 32.0% of shape variance in the nsCLP subgroup. BCLP are indicated in red and UCLP are indicated in blue. There is no separation between the two cohorts, demonstrating that craniofacial skeletal shape variation does not differ in the presence of a unilateral or a bilateral cleft lip/palate.

PCA found that the first 17 out of 30 PCs explain 90% of the craniofacial shape variation within the nsCLP group, with the first three PCs explaining over 41%. A plot of the first two PCs reveals no separation between the unilateral and bilateral cleft individuals along PC1 or PC2 (Figure 4). PC1 explains 20.54% of craniofacial variance and is associated with the angulation of the jaw via changes in the gonial angle as well as the rotation of the condyle. Additionally, it is associated with changes in the cranial base, such as an alteration in the flexion of the posterior cranial base indicated by changes in the positions of basion, opisthion, and the internal acoustic meatus landmarks. PC2 explains 11.47% of the craniofacial variation and the shape changes detected along this axis were the shortening of the length of the cranium, particularly along the posterior cranial base and a downward rotation of the mandible along the positive axis. Additionally, the ANS to PNS distance is shorter and the maxillary plane also rotates downward along the positive axis. PC3 explains an additional 9.25% of the variance. The shape changes along this axis are associated with overall facial width, ranging from wider but shorter skulls along the positive axis to longer but narrower skulls along the negative axis. This PC is also associated with maxillary projection and retrognathism along the negative axis and maxillary hypoplasia and prognathism along the positive axis. Permutation testing found no significant differences between individuals with unilateral and bilateral clefts (PD = 0.308, *p* = 0.183).

## 4. Discussion

The idea that the CLP is superimposed on the genetic and epigenetic growth potential of each individual is not new and may explain the phenotypic variation within each cleft category [59]. Craniofacial morphology remains a complex and highly orchestrated biological process in which the five embryologic prominences surrounding the stomodeum (early mouth) make up the facial primordia, with signaling from bmp, shh, fgf, and wnt pathways directing the overall facial development and shape [60]. Mutations within these same pathways can result in orofacial clefting [61]. In human studies, Boehringer et al. [62] reported two single nucleotide polymorphisms (SNP) associated with nsCLP that also influenced normal variations in nasal width and bizygomatic width, while Indencleef et al. [63] identified 6 SNPs associated with nsCLP which showed significant associations with normal variation of chin, philtrum, nose, and supraorbital ridge development through a meta-analysis of the literature. Thus, is the cleft the sole and primary developmental driver that explains the variations in craniofacial shape? It is possible that genetic perturbations affecting facial width, nasal width, maxillary and mandibular length and prominence, cranial base width and length [61,62,64] may set off a cascade of sequential and potentially subtle developmental variations that ultimately push the boundaries of skeletal development and result in clefting. Studies of the embryonic facial development of cleft susceptible mice strains (A/J, CL/Fr, A/WySn) showed changes in frontonasomaxillary prominence with more medially positioned medial nasal processes and underdevelopment of the maxillary prominence [65,66,67], which is reminiscent of maxillary retrusion and the concave facial profile (skeletal Class III) seen in humans.

Our results demonstrate that the underlying dentofacial deformity or skeletal malocclusion plays a significant role in the overall craniofacial shape and development in patients with CLP. Despite the prevalence of a dentofacial deformity with clefts, the impact of this inherent abnormal growth is poorly understood and not characterized, as demonstrated by the lack of information regarding skeletal deformities in previous cleft studies.

To address these issues, our study used CBCT geometric morphometric analysis for patients with nsCLP and used two curated non-cleft control groups. Most of the previous research studies in patients with nsCLP have evaluated craniofacial morphology with the use of two-dimensional lateral cephalograms. In a few recent studies, three-dimensional CBCT images have also been implemented in order to describe the shape and volumetric effects of orofacial clefts on the maxilla, cranial base, and TMJ. However, to our knowledge, these studies relied on unmatched or age- and sex-matched controls to evaluate the effects of clefts and none included the skeletal malocclusion classification as a variable or as a matching criterion for controls. The inclusion of CBCT images from matched true Class I normal occlusion and skeletal growth (who have no skeletal growth or dentofacial deformities), as well as matched Class II or III skeletal malocclusion subjects as controls allowed us to examine more closely the specific effects of the clefts on craniofacial development in distant sites including the entire maxilla, mandible and cranial base, a combination of factors which has not been previously explored.

The PCA revealed that the first principal component (PC1) provides some separation between the three diagnoses. The dentofacial deformity group falls in the center of the distribution and overlaps with both the unaffected and cleft groups, but the cleft and unaffected control groups fall on opposite sides and separate clearly from each other. The PCA results are supported by the mean shape differences seen in the pairwise Procrustes distance comparisons. Patients with clefts had greater similarity to patients with DFD, as indicated by a smaller Procrustes distance though the maxilla was more hypoplastic and wider than patients with DFD. Additionally, the changes noted in the cleft group are distant from the cleft site, i.e., right and left jugal point and the sagittal position of the mandible. Regarding the mandible, the cleft group has an increased tendency for Class III skeletal relationships (73% of patients), whether it is due to maxillary hypoplasia or mandibular hyperplasia or both. The remaining 27% had Class II skeletal relationship and none of the patients with clefts had a normal relationship of the mandible to skull base. Combined with the measures of overall shape variance, this indicates that the patients with clefts represent a similar but more extreme craniofacial phenotype abnormality than isolated DFD patients when compared to controls. We believe this supports the hypothesis that alterations in craniofacial growth, such as dentofacial deformities, may predispose to additional anomalies, such as a cleft, and increased phenotypic variability.

A study in pairs of monozygotic twins concluded that the presence of a cleft and its surgical repair not only results in a retrusion of the maxilla but also causes alterations in cranial width as well as a rotation of the mandible in the cleft patients. However, despite the surgical repair in one twin, the overall skeletal pattern between the twins was similar, suggesting that the defect of the cleft and skeletal anomalies may have some common genetic influences but that the additional anomaly of a cleft may have independent origins [68]. Chatzistavrou et al. [64] examined the unaffected twin in 33 monozygotic twins discordant for a CLP and compared the craniofacial skeletal morphology of the unaffected twin to a normal control population. They identified craniofacial features that were significantly different in the unaffected twin cohort including reduced nasal width, cranial base width/length ratio, and maxillary width/length ratio but increased cranial base length and suggested these skeletal variations are potential predisposing factors to CLP.

Our analysis of the cleft cohort indicates that the presence of a unilateral versus bilateral cleft did not differentially affect craniofacial shape. However, previous studies involving mixed types of clefts have suggested that there may be some skeletal differences between the two, particularly in the vertical dimensions [69]. Our cleft cohort is small compared to the number of landmarks and this may contribute to the lack of differentiation between the unilateral and bilateral cleft subtypes in the PCA plot, but overall, our results suggest that the presence of any type of cleft is sufficient to alter craniofacial morphology.

The main limitation of this study was that all patients in the nsCLP cohort had previously undergone surgical repair of their CLP, which involves procedures that have been documented in some cases to inhibit normal midface projection and development. While cleft repair can alter facial development, there is literature to suggest that the current standard of care does not have a significant impact on facial projection and growth when done at the appropriate age and with minimal repeat surgery/scarring [70,71]. The cleft care provided to the patients in this study were conducted at two well-established leading craniofacial centers in the United States practicing current standards of care. Despite the potential iatrogenic impact, the effects of cleft repair do not explain the global changes of the maxilla and mandible.

Ideally, to understand the natural effects of clefts on facial development, we would have to include adult patients with unrepaired CLP. However, this is impossible in a developed country. The recruitment and available data of patients with unrepaired cleft defects is very limited due to the known benefits and success of early surgical intervention (standard of care). Additionally, while there are limited studies evaluating unrepaired clefts and the impact on facial development, most of them are case reports and use two-dimensional radiographs that do not provide high resolution and shape data. The results of these studies were variable, with some authors concluding that, in the absence of corrective surgery, the growth potential is similar to normal [72,73,74,75], whereas others suggest that there are inherent morphogenetic abnormalities in the craniofacial morphology of cleft patients [76,77,78,79]. A recent study, Latif et al. [80] examined 39 Indonesian subjects with unrepaired bilateral cleft lip +/− palates using geometric morphometrics on two-dimensional radiographs. They compared their cohort to an age-, sex-, and ethnic- matched controls with no clefts; they did not confirm the skeletal growth patterns of their controls. Despite the limitations of two-dimensional imaging and the lack of a curated control group, they reported an intrinsic growth impairment affecting facial morphology in the unrepaired cleft group, which was distinct from their control group and independent from the effects of surgical repair. Though they did not comment on the highly variable growth pattern of the mandible seen in their graphs, there were clearly distinguishing growth patterns among the unrepaired cleft patients that were distant from the cleft site. Unfortunately, the limitation of two-dimensional radiographs did not permit further delineation of these growth abnormalities.

## 5. Conclusions

Our research suggests that many of the craniofacial changes in the midface and mandible previously associated with the presence of a cleft may in fact be associated with a coexisting dentofacial deformity rather than caused by the cleft itself. Although these have been previously assumed to be the direct result of a cleft on craniofacial development, patients with nsCLP were morphologically similar to the patients with dentofacial deformities but distinct from a true unaffected control group. The type and extent of the underlying skeletal malocclusion present in individuals with a cleft should be considered when determining the effects of a cleft lip and palate on the development of craniofacial structures. We support the evaluation and selection of control groups based on their skeletal malocclusion characteristics for studies involving cleft etiology and growth, as healthy individuals without clefts can have unrelated dentofacial anomalies. Further research into the genetic basis of nsCLP and dentofacial deformities is required to determine whether these developmental and growth perturbations are due to the same genetic, epigenetic, and environmental factors and whether one anomaly influences the risk for the other.

## Figures and Tables

**Table 1:** Sample makeup indicating cohort type, number of individuals, sex, mean age, and ethnicity for all three groups and cleft type (unilateral or bilateral) and laterality (right, left, or bilateral) for nsCLP.

Diagnosis	N	Skeletal Class	N	Sex	Age ± SD	Ethnicity			
						Caucasian (N)	Hispanic (N)	Asian (N)	Unknown (N)
nsCLP	30	Class II	8	17 F, 13 M	17.6 ± 4.2	15	8	4	0
		Class III	22						
DFD	30	Class II	8	17 F, 13 M	22.1 ± 7.5	15	5	6	0
		Class III	22						
Unaffected	60	Class I	60	34 F, 26 M	16.1 ± 2.3	5	2	0	53
Cleft Type	N	Laterality	N						
Bilateral	11	-	11						
Unilateral	19	Right	7						
		Left	12						

## Supplementary Materials

The following are available online at https://www.mdpi.com/2221-3759/8/1/2/s1, Table S1: Mean values and number of standard deviations for SNA, SNB and ANB (angles in degrees) for the study cohorts. Caucasian norms are provided in this table as a reference. However, age, gender and ethnicity matched norms were used for the assessment of each subject., Table S2: List of landmarks and anatomical definitions., Figure S1: Boxplot depicting the trace of the covariance matrix within each cohort. Significant differences were detected in the variance among the cohorts (ANOVA *p* < 0.0001), whereas the nsCLP group had the highest variance, followed by the DFD, while the Normal cohort had the lowest variance.
